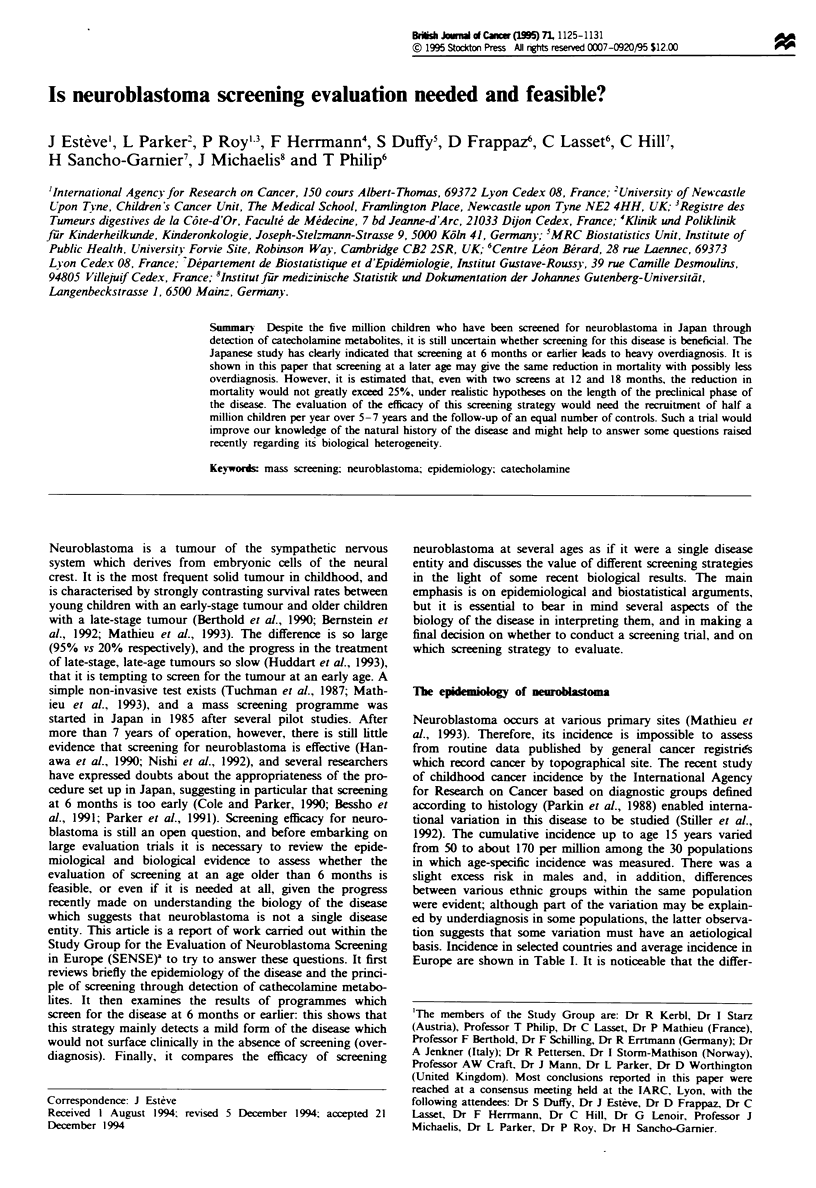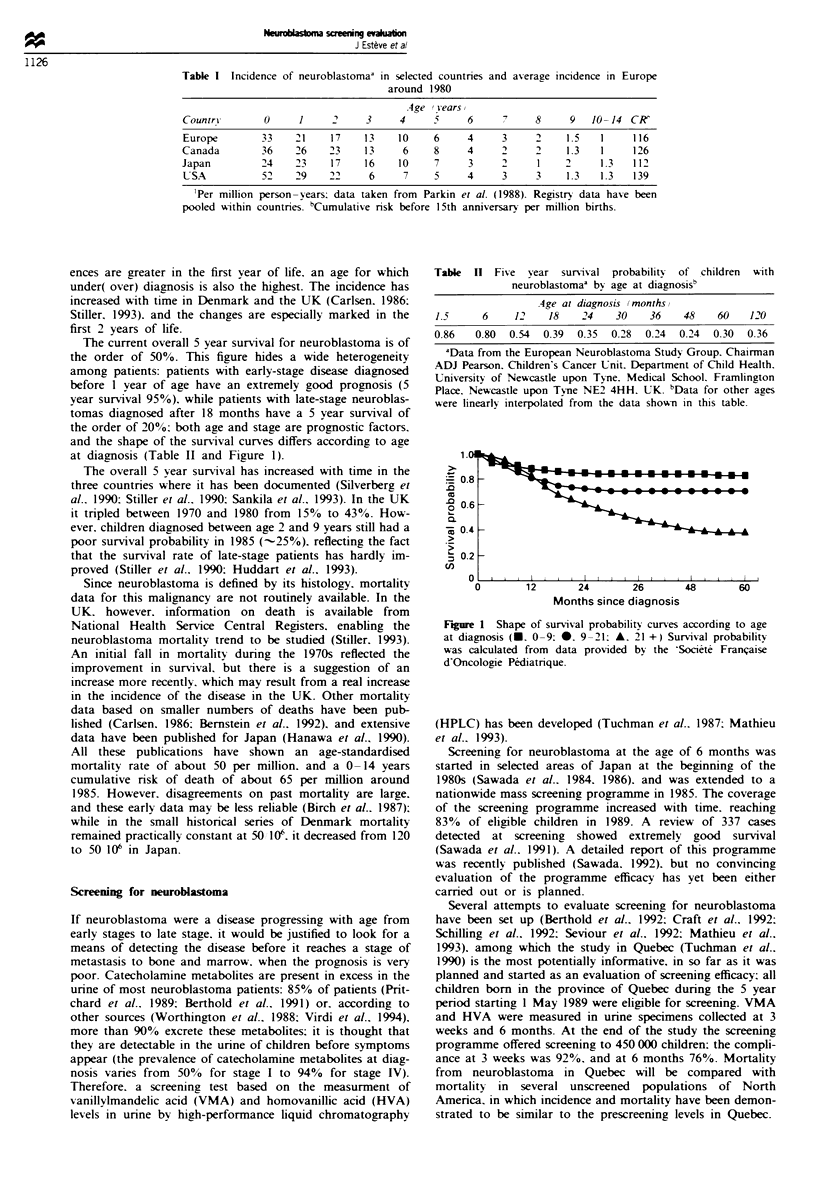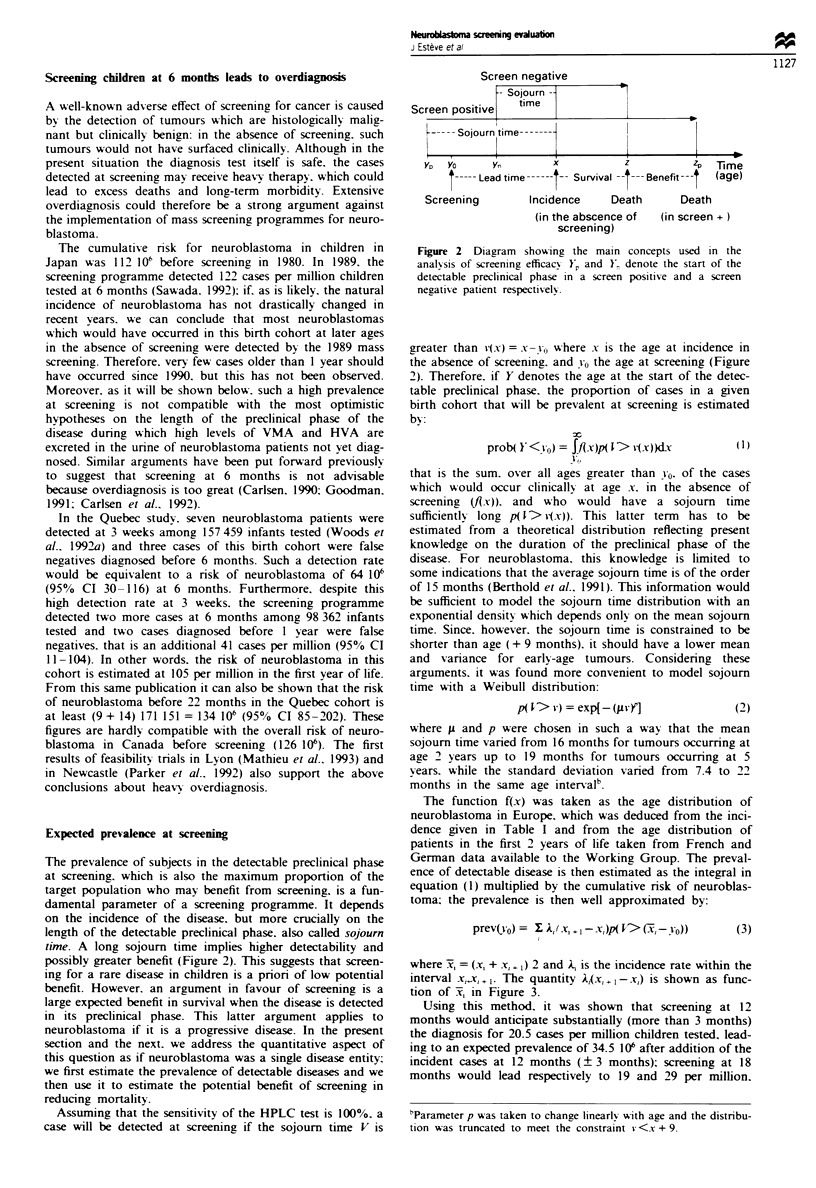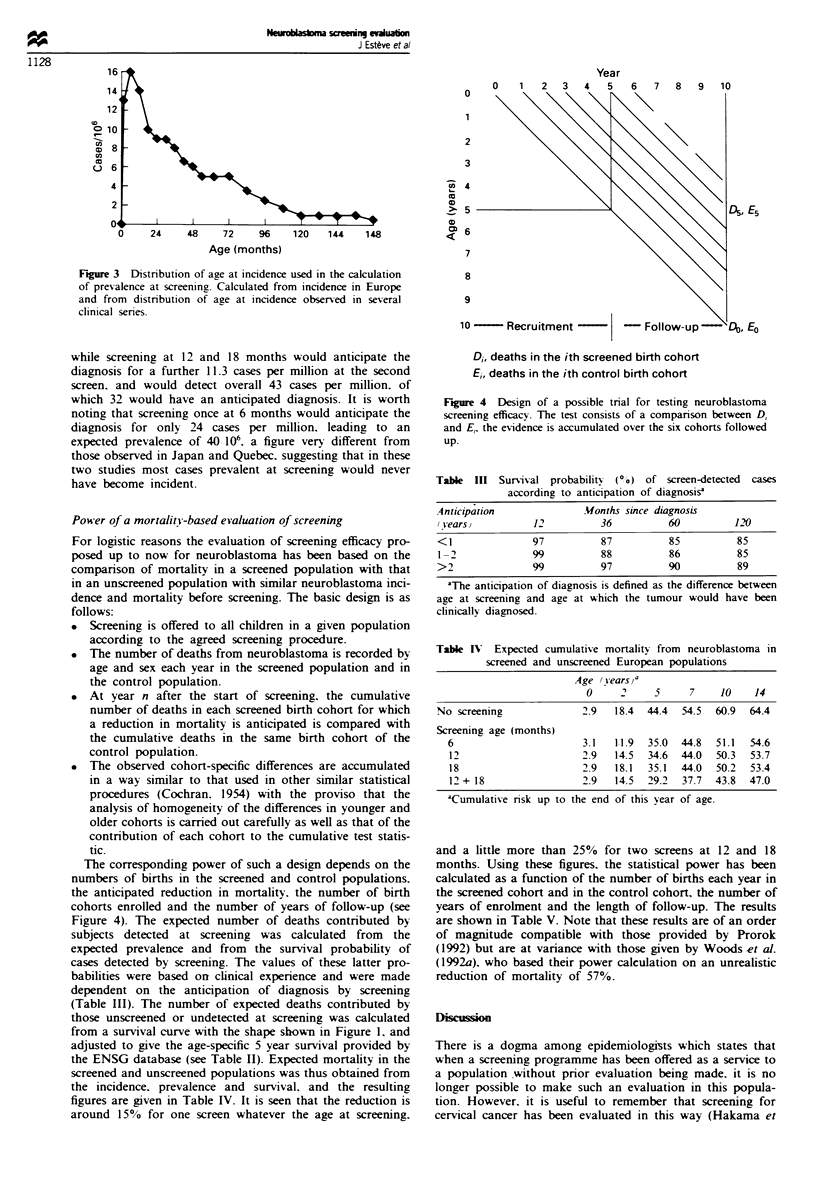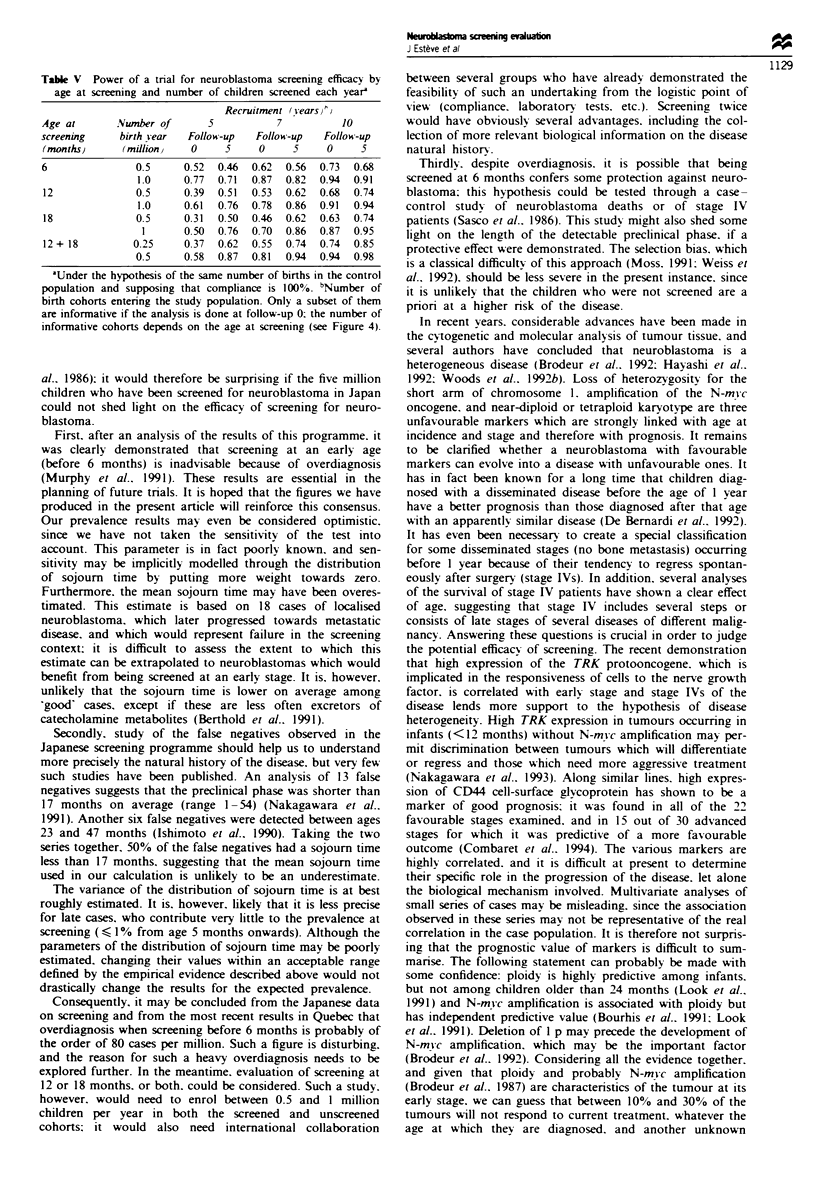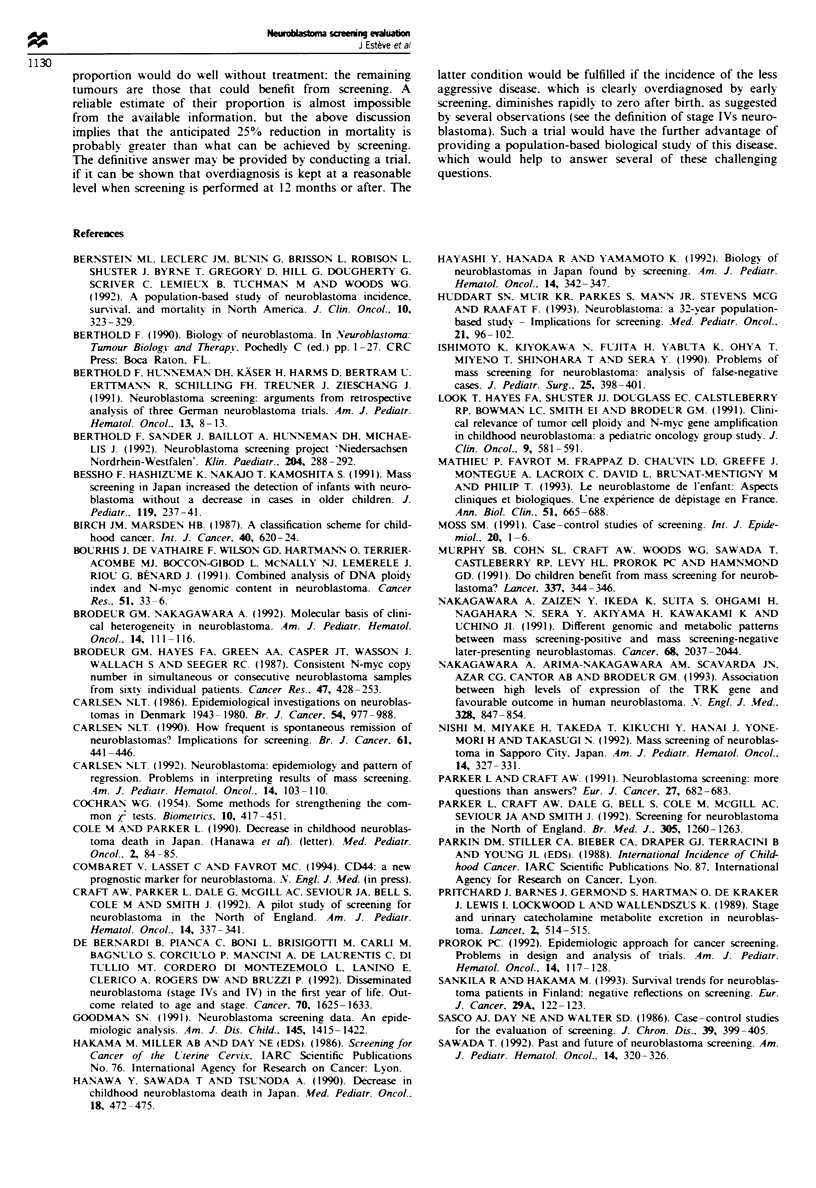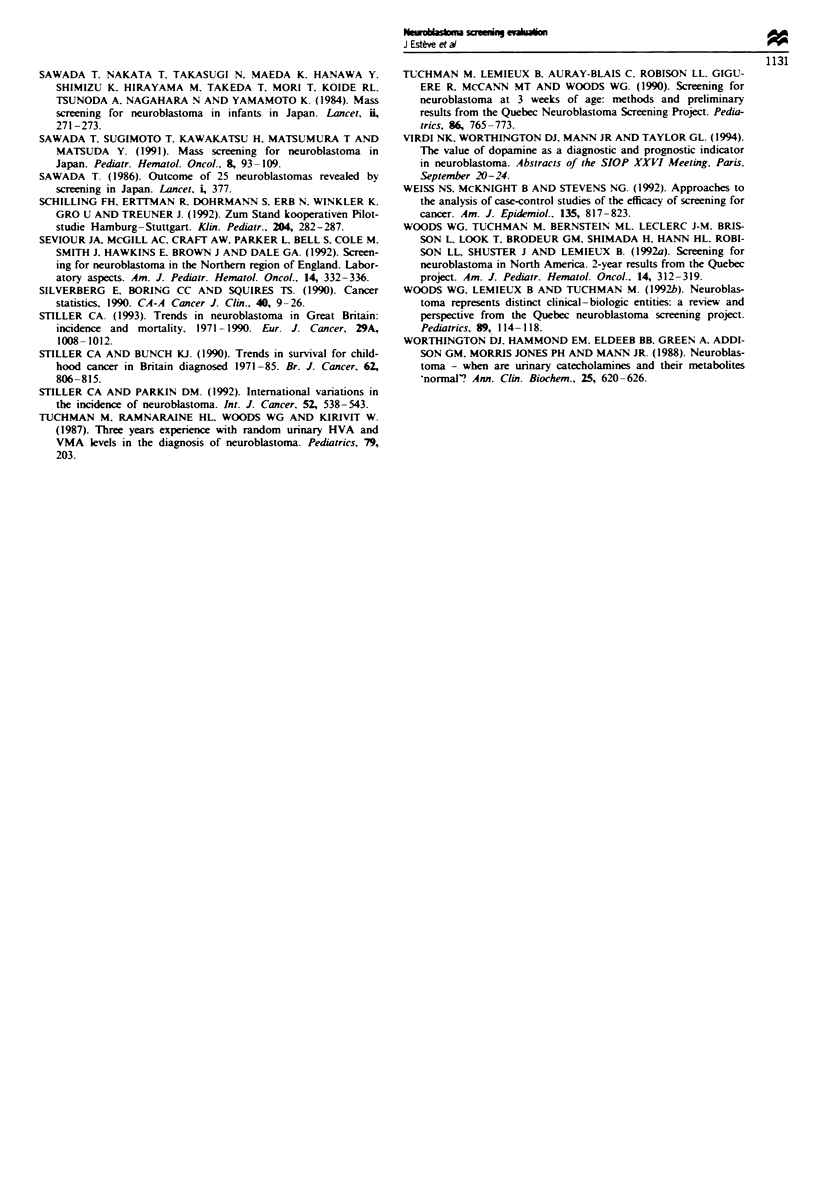# Is neuroblastoma screening evaluation needed and feasible?

**DOI:** 10.1038/bjc.1995.219

**Published:** 1995-06

**Authors:** J. Estève, L. Parker, P. Roy, F. Herrmann, S. Duffy, D. Frappaz, C. Lasset, C. Hill, H. Sancho-Garnier, J. Michaelis

**Affiliations:** International Agency for Research on Cancer, Lyon, France.

## Abstract

Despite the five million children who have been screened for neuroblastoma in Japan through detection of catecholamine metabolites, it is still uncertain whether screening for this disease is beneficial. The Japanese study has clearly indicated that screening at 6 months or earlier leads to heavy overdiagnosis. It is shown in this paper that screening at a later age may give the same reduction in mortality with possibly less overdiagnosis. However, it is estimated that, even with two screens at 12 and 18 months, the reduction in mortality would not greatly exceed 25%, under realistic hypotheses on the length of the preclinical phase of the disease. The evaluation of the efficacy of this screening strategy would need the recruitment of half a million children per year over 5-7 years and the follow-up of an equal number of controls. Such a trial would improve our knowledge of the natural history of the disease and might help to answer some questions raised recently regarding its biological heterogeneity.


British Journal of Cancer (1995) 71, 1125–1131

© 1995 Stockton Press All rights reserved 0007–0920/95 $12.00

### Is neuroblastoma screening evaluation needed and feasible?

J Estève[1], L Parker[2], P Roy[1,3], F Herrmann[4], S Duffy[5], D Frappaz[6], C Lasset[6], C Hill[7], H Sancho-Garnier[7], J Michaelis[8] and T Philip[6]

[1]*International Agency for Research on Cancer, 150 cours Albert-Thomas, 69372 Lyon Cedex 08, France;* [2]*University of Newcastle Upon Tyne, Children's Cancer Unit, The Medical School, Framlington Place, Newcastle upon Tyne NE2 4HH, UK;* [3]*Registre des Tumeurs digestives de la Côte-d'Or, Faculté de Médecine, 7 bd Jeanne-d'Arc, 21033 Dijon Cedex, France;* [4]*Klinik und Poliklinik für Kinderheilkunde, Kinderonkologie, Joseph-Stelzmann-Strasse 9, 5000 Köln 41, Germany;* [5]*MRC Biostatistics Unit, Institute of Public Health, University Forvie Site, Robinson Way, Cambridge CB2 2SR, UK;* [6]*Centre Léon Bérard, 28 rue Laennec, 69373 Lyon Cedex 08, France;* [7]*Département de Biostatistique et d'Epidémiologie, Institut Gustave-Roussy, 39 rue Camille Desmoulins, 94805 Villejuif Cedex, France;* [8]*Institut für medizinische Statistik und Dokumentation der Johannes Gutenberg-Universität, Langenbeckstrasse 1, 6500 Mainz, Germany.*

**Summary** Despite the five million children who have been screened for neuroblastoma in Japan through detection of catecholamine metabolites, it is still uncertain whether screening for this disease is beneficial. The Japanese study has clearly indicated that screening at 6 months or earlier leads to heavy overdiagnosis. It is shown in this paper that screening at a later age may give the same reduction in mortality with possibly less overdiagnosis. However, it is estimated that, even with two screens at 12 and 18 months, the reduction in mortality would not greatly exceed 25%, under realistic hypotheses on the length of the preclinical phase of the disease. The evaluation of the efficacy of this screening strategy would need the recruitment of half a million children per year over 5–7 years and the follow-up of an equal number of controls. Such a trial would improve our knowledge of the natural history of the disease and might help to answer some questions raised recently regarding its biological heterogeneity.

**Keywords:** mass screening; neuroblastoma; epidemiology; catecholamine

Neuroblastoma is a tumour of the sympathetic nervous system which derives from embryonic cells of the neural crest. It is the most frequent solid tumour in childhood, and is characterised by strongly contrasting survival rates between young children with an early-stage tumour and older children with a late-stage tumour (Berthold *et al.*, 1990; Bernstein *et al.*, 1992; Mathieu *et al.*, 1993). The difference is so large (95% *vs* 20% respectively), and the progress in the treatment of late-stage, late-age tumours so slow (Huddart *et al.*, 1993), that it is tempting to screen for the tumour at an early age. A simple non-invasive test exists (Tuchman *et al.*, 1987; Mathieu *et al.*, 1993), and a mass screening programme was started in Japan in 1985 after several pilot studies. After more than 7 years of operation, however, there is still little evidence that screening for neuroblastoma is effective (Hanawa *et al.*, 1990; Nishi *et al.*, 1992), and several researchers have expressed doubts about the appropriateness of the procedure set up in Japan, suggesting in particular that screening at 6 months is too early (Cole and Parker, 1990; Bessho *et al.*, 1991; Parker *et al.*, 1991). Screening efficacy for neuroblastoma is still an open question, and before embarking on large evaluation trials it is necessary to review the epidemiological and biological evidence to assess whether the evaluation of screening at an age older than 6 months is feasible, or even if it is needed at all, given the progress recently made on understanding the biology of the disease which suggests that neuroblastoma is not a single disease entity. This article is a report of work carried out within the Study Group for the Evaluation of Neuroblastoma Screening in Europe (SENSE)[a] to try to answer these questions. It first reviews briefly the epidemiology of the disease and the principle of screening through detection of cathecolamine metabolites. It then examines the results of programmes which screen for the disease at 6 months or earlier: this shows that this strategy mainly detects a mild form of the disease which would not surface clinically in the absence of screening (overdiagnosis). Finally, it compares the efficacy of screening neuroblastoma at several ages as if it were a single disease entity and discusses the value of different screening strategies in the light of some recent biological results. The main emphasis is on epidemiological and biostatistical arguments, but it is essential to bear in mind several aspects of the biology of the disease in interpreting them, and in making a final decision on whether to conduct a screening trial, and on which screening strategy to evaluate.

### The epidemiology of neuroblastoma

Neuroblastoma occurs at various primary sites (Mathieu *et al.*, 1993). Therefore, its incidence is impossible to assess from routine data published by general cancer registries which record cancer by topographical site. The recent study of childhood cancer incidence by the International Agency for Research on Cancer based on diagnostic groups defined according to histology (Parkin *et al.*, 1988) enabled international variation in this disease to be studied (Stiller *et al.*, 1992). The cumulative incidence up to age 15 years varied from 50 to about 170 per million among the 30 populations in which age-specific incidence was measured. There was a slight excess risk in males and, in addition, differences between various ethnic groups within the same population were evident; although part of the variation may be explained by underdiagnosis in some populations, the latter observation suggests that some variation must have an aetiological basis. Incidence in selected countries and average incidence in Europe are shown in Table I. It is noticeable that the differ-

Correspondence: J Estève
Received 1 August 1994; revised 5 December 1994; accepted 21 December 1994

[a]The members of the Study Group are: Dr R Kerbl, Dr I Starz (Austria), Professor T Philip, Dr C Lasset, Dr P Mathieu (France), Professor F Berthold, Dr F Schilling, Dr R Errtmann (Germany); Dr A Jenkner (Italy); Dr R Pettersen, Dr I Storm-Mathison (Norway), Professor AW Craft, Dr J Mann, Dr L Parker, Dr D Worthington (United Kingdom). Most conclusions reported in this paper were reached at a consensus meeting held at the IARC, Lyon, with the following attendees: Dr S Duffy, Dr J Estève, Dr D Frappaz, Dr C Lasset, Dr F Herrmann, Dr C Hill, Dr G Lenoir, Professor J Michaelis, Dr L Parker, Dr P Roy, Dr H Sancho-Garnier.

**Table I** Incidence of neuroblastoma[a] in selected countries and average incidence in Europe around 1980

| | Age (years) | | | | | | | | | | | |
|---|---|---|---|---|---|---|---|---|---|---|---|---|
| *Country* | *0* | *1* | *2* | *3* | *4* | *5* | *6* | *7* | *8* | *9* | *10-14* | *CR*[b] |
| Europe | 33 | 21 | 17 | 13 | 10 | 6 | 4 | 3 | 2 | 1.5 | 1 | 116 |
| Canada | 36 | 26 | 23 | 13 | 6 | 8 | 4 | 2 | 2 | 1.3 | 1 | 126 |
| Japan | 24 | 23 | 17 | 16 | 10 | 7 | 3 | 2 | 1 | 2 | 1.3 | 112 |
| USA | 52 | 29 | 22 | 6 | 7 | 5 | 4 | 3 | 3 | 1.3 | 1.3 | 139 |

[a]Per million person-years; data taken from Parkin *et al.* (1988). Registry data have been pooled within countries. [b]Cumulative risk before 15th anniversary per million births.

ences are greater in the first year of life, an age for which under( over) diagnosis is also the highest. The incidence has increased with time in Denmark and the UK (Carlsen, 1986; Stiller, 1993), and the changes are especially marked in the first 2 years of life.

The current overall 5 year survival for neuroblastoma is of the order of 50%. This figure hides a wide heterogeneity among patients: patients with early-stage disease diagnosed before 1 year of age have an extremely good prognosis (5 year survival 95%), while patients with late-stage neuroblastomas diagnosed after 18 months have a 5 year survival of the order of 20%; both age and stage are prognostic factors, and the shape of the survival curves differs according to age at diagnosis (Table II and Figure 1).

The overall 5 year survival has increased with time in the three countries where it has been documented (Silverberg *et al.*, 1990; Stiller *et al.*, 1990; Sankila *et al.*, 1993). In the UK it tripled between 1970 and 1980 from 15% to 43%. However, children diagnosed between age 2 and 9 years still had a poor survival probability in 1985 (~25%), reflecting the fact that the survival rate of late-stage patients has hardly improved (Stiller *et al.*, 1990; Huddart *et al.*, 1993).

Since neuroblastoma is defined by its histology, mortality data for this malignancy are not routinely available. In the UK, however, information on death is available from National Health Service Central Registers, enabling the neuroblastoma mortality trend to be studied (Stiller, 1993). An initial fall in mortality during the 1970s reflected the improvement in survival, but there is a suggestion of an increase more recently, which may result from a real increase in the incidence of the disease in the UK. Other mortality data based on smaller numbers of deaths have been published (Carlsen, 1986; Bernstein *et al.*, 1992), and extensive data have been published for Japan (Hanawa *et al.*, 1990). All these publications have shown an age-standardised mortality rate of about 50 per million, and a 0-14 years cumulative risk of death of about 65 per million around 1985. However, disagreements on past mortality are large, and these early data may be less reliable (Birch *et al.*, 1987); while in the small historical series of Denmark mortality remained practically constant at 50/10$^6$, it decreased from 120 to 50/10$^6$ in Japan.

**Table II** Five year survival probability of children with neuroblastoma[a] by age at diagnosis[b]

| Age at diagnosis (months) | | | | | | | | | |
|---|---|---|---|---|---|---|---|---|---|
| *1.5* | *6* | *12* | *18* | *24* | *30* | *36* | *48* | *60* | *120* |
| 0.86 | 0.80 | 0.54 | 0.39 | 0.35 | 0.28 | 0.24 | 0.24 | 0.30 | 0.36 |

[a]Data from the European Neuroblastoma Study Group, Chairman ADJ Pearson, Children's Cancer Unit, Department of Child Health, University of Newcastle upon Tyne, Medical School, Framlington Place, Newcastle upon Tyne NE2 4HH, UK. [b]Data for other ages were linearly interpolated from the data shown in this table.

**Figure 1** Shape of survival probability curves according to age at diagnosis (■, 0-9; ●, 9-21; ▲, 21+) Survival probability was calculated from data provided by the 'Société Française d'Oncologie Pédiatrique.

### Screening for neuroblastoma

If neuroblastoma were a disease progressing with age from early stages to late stage, it would be justified to look for a means of detecting the disease before it reaches a stage of metastasis to bone and marrow, when the prognosis is very poor. Catecholamine metabolites are present in excess in the urine of most neuroblastoma patients: 85% of patients (Pritchard *et al.*, 1989; Berthold *et al.*, 1991) or, according to other sources (Worthington *et al.*, 1988; Virdi *et al.*, 1994), more than 90% excrete these metabolites; it is thought that they are detectable in the urine of children before symptoms appear (the prevalence of catecholamine metabolites at diagnosis varies from 50% for stage I to 94% for stage IV). Therefore, a screening test based on the measurment of vanillylmandelic acid (VMA) and homovanillic acid (HVA) levels in urine by high-performance liquid chromatography (HPLC) has been developed (Tuchman *et al.*, 1987; Mathieu *et al.*, 1993).

Screening for neuroblastoma at the age of 6 months was started in selected areas of Japan at the beginning of the 1980s (Sawada *et al.*, 1984, 1986), and was extended to a nationwide mass screening programme in 1985. The coverage of the screening programme increased with time, reaching 83% of eligible children in 1989. A review of 337 cases detected at screening showed extremely good survival (Sawada *et al.*, 1991). A detailed report of this programme was recently published (Sawada, 1992), but no convincing evaluation of the programme efficacy has yet been either carried out or is planned.

Several attempts to evaluate screening for neuroblastoma have been set up (Berthold *et al.*, 1992; Craft *et al.*, 1992; Schilling *et al.*, 1992; Seviour *et al.*, 1992; Mathieu *et al.*, 1993), among which the study in Quebec (Tuchman *et al.*, 1990) is the most potentially informative, in so far as it was planned and started as an evaluation of screening efficacy; all children born in the province of Quebec during the 5 year period starting 1 May 1989 were eligible for screening. VMA and HVA were measured in urine specimens collected at 3 weeks and 6 months. At the end of the study the screening programme offered screening to 450 000 children; the compliance at 3 weeks was 92%, and at 6 months 76%. Mortality from neuroblastoma in Quebec will be compared with mortality in several unscreened populations of North America, in which incidence and mortality have been demonstrated to be similar to the prescreening levels in Quebec.

### Screening children at 6 months leads to overdiagnosis

A well-known adverse effect of screening for cancer is caused by the detection of tumours which are histologically malignant but clinically benign: in the absence of screening, such tumours would not have surfaced clinically. Although in the present situation the diagnosis test itself is safe, the cases detected at screening may receive heavy therapy, which could lead to excess deaths and long-term morbidity. Extensive overdiagnosis could therefore be a strong argument against the implementation of mass screening programmes for neuroblastoma.

The cumulative risk for neuroblastoma in children in Japan was 112 10$^6$ before screening in 1980. In 1989, the screening programme detected 122 cases per million children tested at 6 months (Sawada, 1992); if, as is likely, the natural incidence of neuroblastoma has not drastically changed in recent years, we can conclude that most neuroblastomas which would have occurred in this birth cohort at later ages in the absence of screening were detected by the 1989 mass screening. Therefore, very few cases older than 1 year should have occurred since 1990, but this has not been observed. Moreover, as it will be shown below, such a high prevalence at screening is not compatible with the most optimistic hypotheses on the length of the preclinical phase of the disease during which high levels of VMA and HVA are excreted in the urine of neuroblastoma patients not yet diagnosed. Similar arguments have been put forward previously to suggest that screening at 6 months is not advisable because overdiagnosis is too great (Carlsen, 1990; Goodman, 1991; Carlsen *et al.*, 1992).

In the Quebec study, seven neuroblastoma patients were detected at 3 weeks among 157 459 infants tested (Woods *et al.*, 1992*a*) and three cases of this birth cohort were false negatives diagnosed before 6 months. Such a detection rate would be equivalent to a risk of neuroblastoma of 64 10$^6$ (95% CI 30–116) at 6 months. Furthermore, despite this high detection rate at 3 weeks, the screening programme detected two more cases at 6 months among 98 362 infants tested and two cases diagnosed before 1 year were false negatives, that is an additional 41 cases per million (95% CI 11–104). In other words, the risk of neuroblastoma in this cohort is estimated at 105 per million in the first year of life. From this same publication it can also be shown that the risk of neuroblastoma before 22 months in the Quebec cohort is at least (9 + 14) 171 151 = 134 10$^6$ (95% CI 85–202). These figures are hardly compatible with the overall risk of neuroblastoma in Canada before screening (126 10$^6$). The first results of feasibility trials in Lyon (Mathieu *et al.*, 1993) and in Newcastle (Parker *et al.*, 1992) also support the above conclusions about heavy overdiagnosis.

### Expected prevalence at screening

The prevalence of subjects in the detectable preclinical phase at screening, which is also the maximum proportion of the target population who may benefit from screening, is a fundamental parameter of a screening programme. It depends on the incidence of the disease, but more crucially on the length of the detectable preclinical phase, also called *sojourn time*. A long sojourn time implies higher detectability and possibly greater benefit (Figure 2). This suggests that screening for a rare disease in children is a priori of low potential benefit. However, an argument in favour of screening is a large expected benefit in survival when the disease is detected in its preclinical phase. This latter argument applies to neuroblastoma if it is a progressive disease. In the present section and the next, we address the quantitative aspect of this question as if neuroblastoma was a single disease entity; we first estimate the prevalence of detectable diseases and we then use it to estimate the potential benefit of screening in reducing mortality.

Assuming that the sensitivity of the HPLC test is 100%, a case will be detected at screening if the sojourn time $V$ is

**Figure 2** Diagram showing the main concepts used in the analysis of screening efficacy $Y_p$ and $Y_n$ denote the start of the detectable preclinical phase in a screen positive and a screen negative patient respectively.

greater than $v(x) = x - y_0$ where $x$ is the age at incidence in the absence of screening, and $y_0$ the age at screening (Figure 2). Therefore, if $Y$ denotes the age at the start of the detectable preclinical phase, the proportion of cases in a given birth cohort that will be prevalent at screening is estimated by:

$$\text{prob}(Y < y_0) = \int_{y_0}^{\infty} f(x)p(V > v(x))dx \qquad (1)$$

that is the sum, over all ages greater than $y_0$, of the cases which would occur clinically at age $x$, in the absence of screening ($f(x)$), and who would have a sojourn time sufficiently long $p(V > v(x))$. This latter term has to be estimated from a theoretical distribution reflecting present knowledge on the duration of the preclinical phase of the disease. For neuroblastoma, this knowledge is limited to some indications that the average sojourn time is of the order of 15 months (Berthold *et al.*, 1991). This information would be sufficient to model the sojourn time distribution with an exponential density which depends only on the mean sojourn time. Since, however, the sojourn time is constrained to be shorter than age ( + 9 months), it should have a lower mean and variance for early-age tumours. Considering these arguments, it was found more convenient to model sojourn time with a Weibull distribution:

$$p(V > v) = \exp[-(\mu v)^p] \qquad (2)$$

where $\mu$ and $p$ were chosen in such a way that the mean sojourn time varied from 16 months for tumours occurring at age 2 years up to 19 months for tumours occurring at 5 years, while the standard deviation varied from 7.4 to 22 months in the same age interval[b].

The function f($x$) was taken as the age distribution of neuroblastoma in Europe, which was deduced from the incidence given in Table I and from the age distribution of patients in the first 2 years of life taken from French and German data available to the Working Group. The prevalence of detectable disease is then estimated as the integral in equation (1) multiplied by the cumulative risk of neuroblastoma; the prevalence is then well approximated by:

$$\text{prev}(y_0) = \sum_i \lambda_i(x_{i+1} - x_i)p(V > (\bar{x}_i - y_0)) \qquad (3)$$

where $\bar{x}_i = (x_i + x_{i+1})$ 2 and $\lambda_i$ is the incidence rate within the interval $x_i$-$x_{i+1}$. The quantity $\lambda_i(x_{i+1} - x_i)$ is shown as function of $\bar{x}_i$ in Figure 3.

Using this method, it was shown that screening at 12 months would anticipate substantially (more than 3 months) the diagnosis for 20.5 cases per million children tested, leading to an expected prevalence of 34.5 10$^6$ after addition of the incident cases at 12 months ($\pm$ 3 months); screening at 18 months would lead respectively to 19 and 29 per million,

[b]Parameter $p$ was taken to change linearly with age and the distribution was truncated to meet the constraint $v < x + 9$.

**Figure 3** Distribution of age at incidence used in the calculation of prevalence at screening. Calculated from incidence in Europe and from distribution of age at incidence observed in several clinical series.

while screening at 12 and 18 months would anticipate the diagnosis for a further 11.3 cases per million at the second screen, and would detect overall 43 cases per million, of which 32 would have an anticipated diagnosis. It is worth noting that screening once at 6 months would anticipate the diagnosis for only 24 cases per million, leading to an expected prevalence of 40/10$^6$, a figure very different from those observed in Japan and Quebec, suggesting that in these two studies most cases prevalent at screening would never have become incident.

*Power of a mortality-based evaluation of screening*

For logistic reasons the evaluation of screening efficacy proposed up to now for neuroblastoma has been based on the comparison of mortality in a screened population with that in an unscreened population with similar neuroblastoma incidence and mortality before screening. The basic design is as follows:

- Screening is offered to all children in a given population according to the agreed screening procedure.
- The number of deaths from neuroblastoma is recorded by age and sex each year in the screened population and in the control population.
- At year *n* after the start of screening, the cumulative number of deaths in each screened birth cohort for which a reduction in mortality is anticipated is compared with the cumulative deaths in the same birth cohort of the control population.
- The observed cohort-specific differences are accumulated in a way similar to that used in other similar statistical procedures (Cochran, 1954) with the proviso that the analysis of homogeneity of the differences in younger and older cohorts is carried out carefully as well as that of the contribution of each cohort to the cumulative test statistic.

The corresponding power of such a design depends on the numbers of births in the screened and control populations, the anticipated reduction in mortality, the number of birth cohorts enrolled and the number of years of follow-up (see Figure 4). The expected number of deaths contributed by subjects detected at screening was calculated from the expected prevalence and from the survival probability of cases detected by screening. The values of these latter probabilities were based on clinical experience and were made dependent on the anticipation of diagnosis by screening (Table III). The number of expected deaths contributed by those unscreened or undetected at screening was calculated from a survival curve with the shape shown in Figure 1, and adjusted to give the age-specific 5 year survival provided by the ENSG database (see Table II). Expected mortality in the screened and unscreened populations was thus obtained from the incidence, prevalence and survival, and the resulting figures are given in Table IV. It is seen that the reduction is around 15% for one screen whatever the age at screening,

**Figure 4** Design of a possible trial for testing neuroblastoma screening efficacy. The test consists of a comparison between $D_i$ and $E_i$, the evidence is accumulated over the six cohorts followed up.

**Table III** Survival probability (%) of screen-detected cases according to anticipation of diagnosis[a]

| *Anticipation (years)* | *Months since diagnosis* 12 | 36 | 60 | 120 |
|---|---|---|---|---|
| <1 | 97 | 87 | 85 | 85 |
| 1–2 | 99 | 88 | 86 | 85 |
| >2 | 99 | 97 | 90 | 89 |

[a]The anticipation of diagnosis is defined as the difference between age at screening and age at which the tumour would have been clinically diagnosed.

**Table IV** Expected cumulative mortality from neuroblastoma in screened and unscreened European populations

| | *Age (years)*[a] 0 | 2 | 5 | 7 | 10 | 14 |
|---|---|---|---|---|---|---|
| No screening | 2.9 | 18.4 | 44.4 | 54.5 | 60.9 | 64.4 |
| Screening age (months) | | | | | | |
| 6 | 3.1 | 11.9 | 35.0 | 44.8 | 51.1 | 54.6 |
| 12 | 2.9 | 14.5 | 34.6 | 44.0 | 50.3 | 53.7 |
| 18 | 2.9 | 18.1 | 35.1 | 44.0 | 50.2 | 53.4 |
| 12 + 18 | 2.9 | 14.5 | 29.2 | 37.7 | 43.8 | 47.0 |

[a]Cumulative risk up to the end of this year of age.

and a little more than 25% for two screens at 12 and 18 months. Using these figures, the statistical power has been calculated as a function of the number of births each year in the screened cohort and in the control cohort, the number of years of enrolment and the length of follow-up. The results are shown in Table V. Note that these results are of an order of magnitude compatible with those provided by Prorok (1992) but are at variance with those given by Woods *et al.* (1992*a*), who based their power calculation on an unrealistic reduction of mortality of 57%.

### Discussion

There is a dogma among epidemiologists which states that when a screening programme has been offered as a service to a population without prior evaluation being made, it is no longer possible to make such an evaluation in this population. However, it is useful to remember that screening for cervical cancer has been evaluated in this way (Hakama *et*

**Table V** Power of a trial for neuroblastoma screening efficacy by age at screening and number of children screened each year[a]

| Age at screening (months) | Number of birth year (million) | Recruitment (years)[b] 5 Follow-up 0 | 5 Follow-up 5 | 7 Follow-up 0 | 7 Follow-up 5 | 10 Follow-up 0 | 10 Follow-up 5 |
|---|---|---|---|---|---|---|---|
| 6 | 0.5 | 0.52 | 0.46 | 0.62 | 0.56 | 0.73 | 0.68 |
| | 1.0 | 0.77 | 0.71 | 0.87 | 0.82 | 0.94 | 0.91 |
| 12 | 0.5 | 0.39 | 0.51 | 0.53 | 0.62 | 0.68 | 0.74 |
| | 1.0 | 0.61 | 0.76 | 0.78 | 0.86 | 0.91 | 0.94 |
| 18 | 0.5 | 0.31 | 0.50 | 0.46 | 0.62 | 0.63 | 0.74 |
| | 1 | 0.50 | 0.76 | 0.70 | 0.86 | 0.87 | 0.95 |
| 12 + 18 | 0.25 | 0.37 | 0.62 | 0.55 | 0.74 | 0.74 | 0.85 |
| | 0.5 | 0.58 | 0.87 | 0.81 | 0.94 | 0.94 | 0.98 |

[a]Under the hypothesis of the same number of births in the control population and supposing that compliance is 100%. [b]Number of birth cohorts entering the study population. Only a subset of them are informative if the analysis is done at follow-up 0; the number of informative cohorts depends on the age at screening (see Figure 4).

*al.*, 1986); it would therefore be surprising if the five million children who have been screened for neuroblastoma in Japan could not shed light on the efficacy of screening for neuroblastoma.

First, after an analysis of the results of this programme, it was clearly demonstrated that screening at an early age (before 6 months) is inadvisable because of overdiagnosis (Murphy *et al.*, 1991). These results are essential in the planning of future trials. It is hoped that the figures we have produced in the present article will reinforce this consensus. Our prevalence results may even be considered optimistic, since we have not taken the sensitivity of the test into account. This parameter is in fact poorly known, and sensitivity may be implicitly modelled through the distribution of sojourn time by putting more weight towards zero. Furthermore, the mean sojourn time may have been overestimated. This estimate is based on 18 cases of localised neuroblastoma, which later progressed towards metastatic disease, and which would represent failure in the screening context; it is difficult to assess the extent to which this estimate can be extrapolated to neuroblastomas which would benefit from being screened at an early stage. It is, however, unlikely that the sojourn time is lower on average among 'good' cases, except if these are less often excretors of catecholamine metabolites (Berthold *et al.*, 1991).

Secondly, study of the false negatives observed in the Japanese screening programme should help us to understand more precisely the natural history of the disease, but very few such studies have been published. An analysis of 13 false negatives suggests that the preclinical phase was shorter than 17 months on average (range 1–54) (Nakagawara *et al.*, 1991). Another six false negatives were detected between ages 23 and 47 months (Ishimoto *et al.*, 1990). Taking the two series together, 50% of the false negatives had a sojourn time less than 17 months, suggesting that the mean sojourn time used in our calculation is unlikely to be an underestimate.

The variance of the distribution of sojourn time is at best roughly estimated. It is, however, likely that it is less precise for late cases, who contribute very little to the prevalence at screening ($\leqslant 1\%$ from age 5 months onwards). Although the parameters of the distribution of sojourn time may be poorly estimated, changing their values within an acceptable range defined by the empirical evidence described above would not drastically change the results for the expected prevalence.

Consequently, it may be concluded from the Japanese data on screening and from the most recent results in Quebec that overdiagnosis when screening before 6 months is probably of the order of 80 cases per million. Such a figure is disturbing, and the reason for such a heavy overdiagnosis needs to be explored further. In the meantime, evaluation of screening at 12 or 18 months, or both, could be considered. Such a study, however, would need to enrol between 0.5 and 1 million children per year in both the screened and unscreened cohorts; it would also need international collaboration between several groups who have already demonstrated the feasibility of such an undertaking from the logistic point of view (compliance, laboratory tests, etc.). Screening twice would have obviously several advantages, including the collection of more relevant biological information on the disease natural history.

Thirdly, despite overdiagnosis, it is possible that being screened at 6 months confers some protection against neuroblastoma; this hypothesis could be tested through a case–control study of neuroblastoma deaths or of stage IV patients (Sasco *et al.*, 1986). This study might also shed some light on the length of the detectable preclinical phase, if a protective effect were demonstrated. The selection bias, which is a classical difficulty of this approach (Moss, 1991; Weiss *et al.*, 1992), should be less severe in the present instance, since it is unlikely that the children who were not screened are a priori at a higher risk of the disease.

In recent years, considerable advances have been made in the cytogenetic and molecular analysis of tumour tissue, and several authors have concluded that neuroblastoma is a heterogeneous disease (Brodeur *et al.*, 1992; Hayashi *et al.*, 1992; Woods *et al.*, 1992*b*). Loss of heterozygosity for the short arm of chromosome 1, amplification of the N-*myc* oncogene, and near-diploid or tetraploid karyotype are three unfavourable markers which are strongly linked with age at incidence and stage and therefore with prognosis. It remains to be clarified whether a neuroblastoma with favourable markers can evolve into a disease with unfavourable ones. It has in fact been known for a long time that children diagnosed with a disseminated disease before the age of 1 year have a better prognosis than those diagnosed after that age with an apparently similar disease (De Bernardi *et al.*, 1992). It has even been necessary to create a special classification for some disseminated stages (no bone metastasis) occurring before 1 year because of their tendency to regress spontaneously after surgery (stage IVs). In addition, several analyses of the survival of stage IV patients have shown a clear effect of age, suggesting that stage IV includes several steps or consists of late stages of several diseases of different malignancy. Answering these questions is crucial in order to judge the potential efficacy of screening. The recent demonstration that high expression of the *TRK* protooncogene, which is implicated in the responsiveness of cells to the nerve growth factor, is correlated with early stage and stage IVs of the disease lends more support to the hypothesis of disease heterogeneity. High *TRK* expression in tumours occurring in infants ($<12$ months) without N-*myc* amplification may permit discrimination between tumours which will differentiate or regress and those which need more aggressive treatment (Nakagawara *et al.*, 1993). Along similar lines, high expression of CD44 cell-surface glycoprotein has shown to be a marker of good prognosis; it was found in all of the 22 favourable stages examined, and in 15 out of 30 advanced stages for which it was predictive of a more favourable outcome (Combaret *et al.*, 1994). The various markers are highly correlated, and it is difficult at present to determine their specific role in the progression of the disease, let alone the biological mechanism involved. Multivariate analyses of small series of cases may be misleading, since the association observed in these series may not be representative of the real correlation in the case population. It is therefore not surprising that the prognostic value of markers is difficult to summarise. The following statement can probably be made with some confidence: ploidy is highly predictive among infants, but not among children older than 24 months (Look *et al.*, 1991) and N-*myc* amplification is associated with ploidy but has independent predictive value (Bourhis *et al.*, 1991; Look *et al.*, 1991). Deletion of 1 p may precede the development of N-*myc* amplification, which may be the important factor (Brodeur *et al.*, 1992). Considering all the evidence together, and given that ploidy and probably N-*myc* amplification (Brodeur *et al.*, 1987) are characteristics of the tumour at its early stage, we can guess that between 10% and 30% of the tumours will not respond to current treatment, whatever the age at which they are diagnosed, and another unknown proportion would do well without treatment: the remaining tumours are those that could benefit from screening. A reliable estimate of their proportion is almost impossible from the available information, but the above discussion implies that the anticipated 25% reduction in mortality is probably greater than what can be achieved by screening. The definitive answer may be provided by conducting a trial, if it can be shown that overdiagnosis is kept at a reasonable level when screening is performed at 12 months or after. The latter condition would be fulfilled if the incidence of the less aggressive disease, which is clearly overdiagnosed by early screening, diminishes rapidly to zero after birth, as suggested by several observations (see the definition of stage IVs neuroblastoma). Such a trial would have the further advantage of providing a population-based biological study of this disease, which would help to answer several of these challenging questions.

### References

BERNSTEIN ML, LECLERC JM, BUNIN G, BRISSON L, ROBISON L, SHUSTER J, BYRNE T, GREGORY D, HILL G, DOUGHERTY G, SCRIVER C, LEMIEUX B, TUCHMAN M AND WOODS WG. (1992). A population-based study of neuroblastoma incidence, survival, and mortality in North America. *J. Clin. Oncol.*, **10**, 323–329.

BERTHOLD F. (1990). Biology of neuroblastoma. In *Neuroblastoma: Tumour Biology and Therapy*, Pochedly C (ed.) pp. 1–27. CRC Press: Boca Raton, FL.

BERTHOLD F, HUNNEMAN DH, KÄSER H, HARMS D, BERTRAM U, ERTTMANN R, SCHILLING FH, TREUNER J, ZIESCHANG J. (1991). Neuroblastoma screening: arguments from retrospective analysis of three German neuroblastoma trials. *Am. J. Pediatr. Hematol. Oncol.*, **13**, 8–13.

BERTHOLD F, SANDER J, BAILLOT A, HUNNEMAN DH, MICHAELIS J. (1992). Neuroblastoma screening project 'Niedersachsen Nordrhein-Westfalen'. *Klin. Paediatr.*, **204**, 288–292.

BESSHO F, HASHIZUME K, NAKAJO T, KAMOSHITA S. (1991). Mass screening in Japan increased the detection of infants with neuroblastoma without a decrease in cases in older children. *J. Pediatr.*, **119**, 237–41.

BIRCH JM, MARSDEN HB. (1987). A classification scheme for childhood cancer. *Int. J. Cancer*, **40**, 620–24.

BOURHIS J, DE VATHAIRE F, WILSON GD, HARTMANN O, TERRIER-ACOMBE MJ, BOCCON-GIBOD L, MCNALLY NJ, LEMERELE J, RIOU G, BÉNARD J. (1991). Combined analysis of DNA ploidy index and N-myc genomic content in neuroblastoma. *Cancer Res.*, **51**, 33–6.

BRODEUR GM, NAKAGAWARA A. (1992). Molecular basis of clinical heterogeneity in neuroblastoma. *Am. J. Pediatr. Hematol. Oncol.*, **14**, 111–116.

BRODEUR GM, HAYES FA, GREEN AA, CASPER JT, WASSON J, WALLACH S AND SEEGER RC. (1987). Consistent N-myc copy number in simultaneous or consecutive neuroblastoma samples from sixty individual patients. *Cancer Res.*, **47**, 428–253.

CARLSEN NLT. (1986). Epidemiological investigations on neuroblastomas in Denmark 1943–1980. *Br. J. Cancer*, **54**, 977–988.

CARLSEN NLT. (1990). How frequent is spontaneous remission of neuroblastomas? Implications for screening. *Br. J. Cancer*, **61**, 441–446.

CARLSEN NLT. (1992). Neuroblastoma: epidemiology and pattern of regression. Problems in interpreting results of mass screening. *Am. J. Pediatr. Hematol. Oncol.*, **14**, 103–110.

COCHRAN WG. (1954). Some methods for strengthening the common $\chi^2$ tests. *Biometrics*, **10**, 417–451.

COLE M AND PARKER L. (1990). Decrease in childhood neuroblastoma death in Japan. (Hanawa *et al*). (letter). *Med. Pediatr. Oncol.*, **2**, 84–85.

COMBARET V, LASSET C AND FAVROT MC. (1994). CD44: a new prognostic marker for neuroblastoma. *N. Engl. J. Med.* (in press).

CRAFT AW, PARKER L, DALE G, MCGILL AC, SEVIOUR JA, BELL S, COLE M AND SMITH J. (1992). A pilot study of screening for neuroblastoma in the North of England. *Am. J. Pediatr. Hematol. Oncol.*, **14**, 337–341.

DE BERNARDI B, PIANCA C, BONI L, BRISIGOTTI M, CARLI M, BAGNULO S, CORCIULO P, MANCINI A, DE LAURENTIS C, DI TULLIO MT, CORDERO DI MONTEZEMOLO L, LANINO E, CLERICO A, ROGERS DW AND BRUZZI P. (1992). Disseminated neuroblastoma (stage IVs and IV) in the first year of life. Outcome related to age and stage. *Cancer*, **70**, 1625–1633.

GOODMAN SN. (1991). Neuroblastoma screening data. An epidemiologic analysis. *Am. J. Dis. Child.*, **145**, 1415–1422.

HAKAMA M, MILLER AB AND DAY NE (EDS). (1986). *Screening for Cancer of the Uterine Cervix*, IARC Scientific Publications No. 76. International Agency for Research on Cancer: Lyon.

HANAWA Y, SAWADA T AND TSUNODA A. (1990). Decrease in childhood neuroblastoma death in Japan. *Med. Pediatr. Oncol.*, **18**, 472–475.

HAYASHI Y, HANADA R AND YAMAMOTO K. (1992). Biology of neuroblastomas in Japan found by screening. *Am. J. Pediatr. Hematol. Oncol.*, **14**, 342–347.

HUDDART SN, MUIR KR, PARKES S, MANN JR, STEVENS MCG AND RAAFAT F. (1993). Neuroblastoma: a 32-year population-based study – Implications for screening. *Med. Pediatr. Oncol.*, **21**, 96–102.

ISHIMOTO K, KIYOKAWA N, FUJITA H, YABUTA K, OHYA T, MIYENO T, SHINOHARA T AND SERA Y. (1990). Problems of mass screening for neuroblastoma: analysis of false-negative cases. *J. Pediatr. Surg.*, **25**, 398–401.

LOOK T, HAYES FA, SHUSTER JJ, DOUGLASS EC, CALSTLEBERRY RP, BOWMAN LC, SMITH EI AND BRODEUR GM. (1991). Clinical relevance of tumor cell ploidy and N-myc gene amplification in childhood neuroblastoma: a pediatric oncology group study. *J. Clin. Oncol.*, **9**, 581–591.

MATHIEU P, FAVROT M, FRAPPAZ D, CHAUVIN LD, GREFFE J, MONTEGUE A, LACROIX C, DAVID L, BRUNAT-MENTIGNY M AND PHILIP T. (1993). Le neuroblastome de l'enfant: Aspects cliniques et biologiques. Une expérience de dépistage en France. *Ann. Biol. Clin.*, **51**, 665–688.

MOSS SM. (1991). Case–control studies of screening. *Int. J. Epidemiol.*, **20**, 1–6.

MURPHY SB, COHN SL, CRAFT AW, WOODS WG, SAWADA T, CASTLEBERRY RP, LEVY HL, PROROK PC AND HAMNMOND GD. (1991). Do children benefit from mass screening for neuroblastoma? *Lancet*, **337**, 344–346.

NAKAGAWARA A, ZAIZEN Y, IKEDA K, SUITA S, OHGAMI H, NAGAHARA N, SERA Y, AKIYAMA H, KAWAKAMI K AND UCHINO JI. (1991). Different genomic and metabolic patterns between mass screening-positive and mass screening-negative later-presenting neuroblastomas. *Cancer*, **68**, 2037–2044.

NAKAGAWARA A, ARIMA-NAKAGAWARA AM, SCAVARDA JN, AZAR CG, CANTOR AB AND BRODEUR GM. (1993). Association between high levels of expression of the TRK gene and favourable outcome in human neuroblastoma. *N. Engl. J. Med.*, **328**, 847–854.

NISHI M, MIYAKE H, TAKEDA T, KIKUCHI Y, HANAI J, YONEMORI H AND TAKASUGI N. (1992). Mass screening of neuroblastoma in Sapporo City, Japan. *Am. J. Pediatr. Hematol. Oncol.*, **14**, 327–331.

PARKER L AND CRAFT AW. (1991). Neuroblastoma screening: more questions than answers? *Eur. J. Cancer*, **27**, 682–683.

PARKER L, CRAFT AW, DALE G, BELL S, COLE M, MCGILL AC, SEVIOUR JA AND SMITH J. (1992). Screening for neuroblastoma in the North of England. *Br. Med. J.*, **305**, 1260–1263.

PARKIN DM, STILLER CA, BIEBER CA, DRAPER GJ, TERRACINI B AND YOUNG JL (EDS). (1988). *International Incidence of Childhood Cancer*. IARC Scientific Publications No. 87. International Agency for Research on Cancer, Lyon.

PRITCHARD J, BARNES J, GERMOND S, HARTMAN O, DE KRAKER J, LEWIS I, LOCKWOOD L AND WALLENDSZUS K. (1989). Stage and urinary catecholamine metabolite excretion in neuroblastoma. *Lancet*, **2**, 514–515.

PROROK PC. (1992). Epidemiologic approach for cancer screening. Problems in design and analysis of trials. *Am. J. Pediatr. Hematol. Oncol.*, **14**, 117–128.

SANKILA R AND HAKAMA M. (1993). Survival trends for neuroblastoma patients in Finland: negative reflections on screening. *Eur. J. Cancer*, **29A**, 122–123.

SASCO AJ, DAY NE AND WALTER SD. (1986). Case–control studies for the evaluation of screening. *J. Chron. Dis.*, **39**, 399–405.

SAWADA T. (1992). Past and future of neuroblastoma screening. *Am. J. Pediatr. Hematol. Oncol.*, **14**, 320–326.

SAWADA T, NAKATA T, TAKASUGI N, MAEDA K, HANAWA Y, SHIMIZU K, HIRAYAMA M, TAKEDA T, MORI T, KOIDE RL, TSUNODA A, NAGAHARA N AND YAMAMOTO K. (1984). Mass screening for neuroblastoma in infants in Japan. *Lancet*, **ii**, 271-273.

SAWADA T, SUGIMOTO T, KAWAKATSU H, MATSUMURA T AND MATSUDA Y. (1991). Mass screening for neuroblastoma in Japan. *Pediatr. Hematol. Oncol.*, **8**, 93-109.

SAWADA T. (1986). Outcome of 25 neuroblastomas revealed by screening in Japan. *Lancet*, **i**, 377.

SCHILLING FH, ERTTMAN R, DOHRMANN S, ERB N, WINKLER K, GRO U AND TREUNER J. (1992). Zum Stand kooperativen Pilotstudie Hamburg-Stuttgart. *Klin. Pediatr.*, **204**, 282-287.

SEVIOUR JA, McGILL AC, CRAFT AW, PARKER L, BELL S, COLE M, SMITH J, HAWKINS E, BROWN J AND DALE GA. (1992). Screening for neuroblastoma in the Northern region of England. Laboratory aspects. *Am. J. Pediatr. Hematol. Oncol.*, **14**, 332-336.

SILVERBERG E, BORING CC AND SQUIRES TS. (1990). Cancer statistics, 1990. *CA-A Cancer J. Clin.*, **40**, 9-26.

STILLER CA. (1993). Trends in neuroblastoma in Great Britain: incidence and mortality, 1971-1990. *Eur. J. Cancer*, **29A**, 1008-1012.

STILLER CA AND BUNCH KJ. (1990). Trends in survival for childhood cancer in Britain diagnosed 1971-85. *Br. J. Cancer*, **62**, 806-815.

STILLER CA AND PARKIN DM. (1992). International variations in the incidence of neuroblastoma. *Int. J. Cancer*, **52**, 538-543.

TUCHMAN M, RAMNARAINE HL, WOODS WG AND KIRIVIT W. (1987). Three years experience with random urinary HVA and VMA levels in the diagnosis of neuroblastoma. *Pediatrics*, **79**, 203.

TUCHMAN M, LEMIEUX B, AURAY-BLAIS C, ROBISON LL, GIGUERE R, McCANN MT AND WOODS WG. (1990). Screening for neuroblastoma at 3 weeks of age: methods and preliminary results from the Quebec Neuroblastoma Screening Project. *Pediatrics*, **86**, 765-773.

VIRDI NK, WORTHINGTON DJ, MANN JR AND TAYLOR GL. (1994). The value of dopamine as a diagnostic and prognostic indicator in neuroblastoma. *Abstracts of the SIOP XXVI Meeting, Paris, September 20-24.*

WEISS NS, McKNIGHT B AND STEVENS NG. (1992). Approaches to the analysis of case-control studies of the efficacy of screening for cancer. *Am. J. Epidemiol.*, **135**, 817-823.

WOODS WG, TUCHMAN M, BERNSTEIN ML, LECLERC J-M, BRISSON L, LOOK T, BRODEUR GM, SHIMADA H, HANN HL, ROBISON LL, SHUSTER J AND LEMIEUX B. (1992*a*). Screening for neuroblastoma in North America. 2-year results from the Quebec project. *Am. J. Pediatr. Hematol. Oncol.*, **14**, 312-319.

WOODS WG, LEMIEUX B AND TUCHMAN M. (1992*b*). Neuroblastoma represents distinct clinical-biologic entities: a review and perspective from the Quebec neuroblastoma screening project. *Pediatrics*, **89**, 114-118.

WORTHINGTON DJ, HAMMOND EM, ELDEEB BB, GREEN A, ADDISON GM, MORRIS JONES PH AND MANN JR. (1988). Neuroblastoma - when are urinary catecholamines and their metabolites 'normal'? *Ann. Clin. Biochem.*, **25**, 620-626.