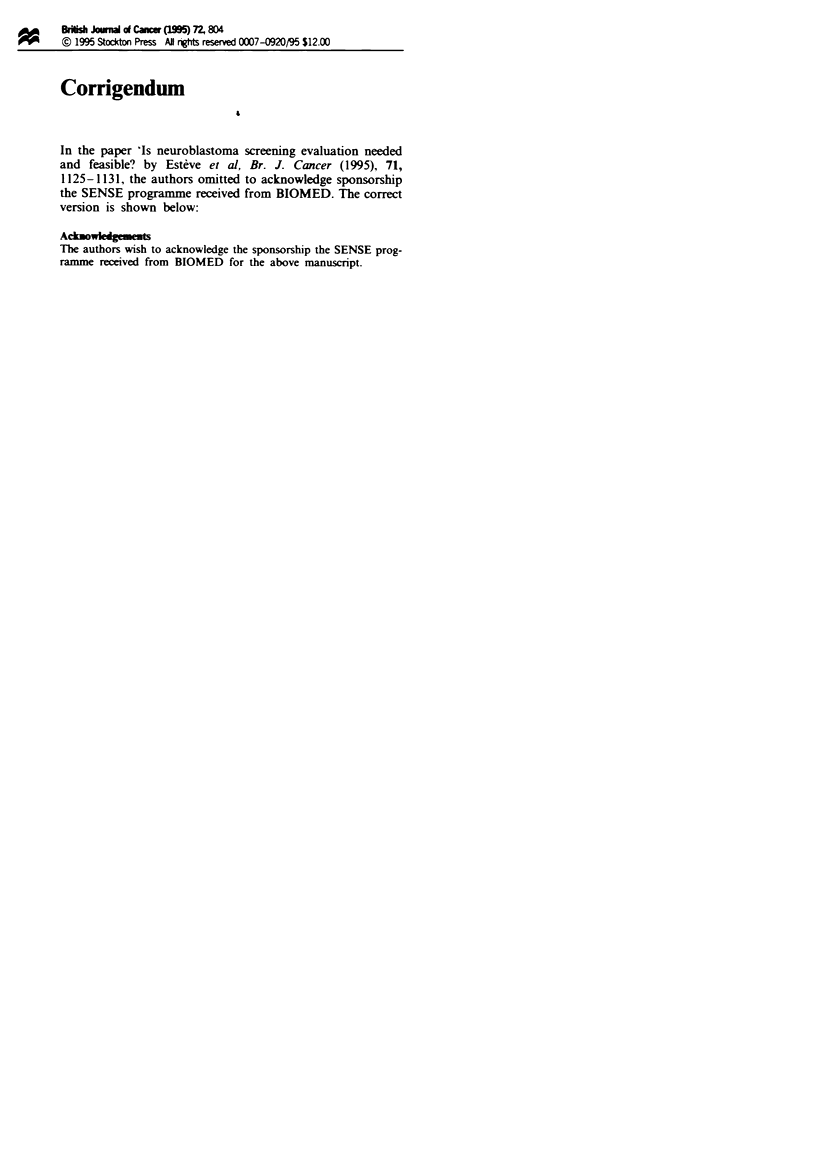# Corrigendum

**Published:** 1995-09

**Authors:** 


British Journal of Cancer (1995) 72, 804

© 1995 Stockton Press All rights reserved 0007-0920/95 $12.00

### Corrigendum

In the paper 'Is neuroblastoma screening evaluation needed and feasible? by Estève *et al*, *Br. J. Cancer* (1995), **71**, 1125–1131, the authors omitted to acknowledge sponsorship the SENSE programme received from BIOMED. The correct version is shown below:

**Acknowledgements**
The authors wish to acknowledge the sponsorship the SENSE programme received from BIOMED for the above manuscript.